# Supplementary material for: Structural basis for higher-order DNA binding by a bacterial transcriptional regulator
Source: PLoS Genet. 2025 Jun 27;21(6):e1011749. doi: 10.1371/journal.pgen.1011749 (PMC12204516; doi:10.1371/journal.pgen.1011749)
Supplement: S1 Table — (DOCX) [file pgen.1011749.s008.docx]

**S1 Table. Promoter DNA sequences**

| **Name** | **DNA sequence (5’-3’)** |
| --- | --- |
| P_XR_ | AG**TTTTTGGGGCCGCTTTGCGGCCCTGGCA**ATGCTGGCGGTCA**ACCTGGCAAAGCCCTGGCTTCGACCGCCCGACCCGGCAAACTGGCAATGACCCGGCA**TATACCG**CATA*CTTG*TCGGCAAATGCCGA*AAAG*GAGC**ATGCC**ATG**CTTGCCGAAGTGCTTCGCGACAACGGT |
| P_XR_∆S1 | TGGCGGTCA**ACCTGGCAAAGCCCTGGCTTCGACCGCCCGACCCGGCAAACTGGCAATGACCCGGCA**TATACCG**CATA*CTTG*TCGGCAAATGCCGA*AAAG*GAGC**ATGCC**ATG**CTTGCCGAAGTGCTTCGCGACAACGGT |
| P_XR_∆S1-2 | **CGACCCGGCAAACTGGCAATGACCCGGCA**TATACCG**CATA*CTTG*TCGGCAAATGCCGA*AAAG*GAGC**ATGCC**ATG**CTTGCCGAAGTGCTTCGCGACAACGGT |
| P_XR_∆S1-3 | ATACCG**CATA*CTTG*TCGGCAAATGCCGA*AAAG*GAGC**ATGCC**ATG**CTTGCCGAAGTGCTTCGCGACAACGGT |
| P_XR_∆S4 | AG**TTTTTGGGGCCGCTTTGCGGCCCTGGCA**ATGCTGGCGGTCA**ACCTGGCAAAGCCCTGGCTTCGACCGCCCGACCCGGCAAACTGGCAATGACCCGGCA**TATACCG---ATGCC**ATG**CTTGCCGAAGTGCTTCGCGACAACGGT |
| P_XR_∆3-4 | AG**TTTTTGGGGCCGCTTTGCGGCCCTGGCA**ATGCTGGCGGTCA**ACCTGGCAAAGCCCTGGCTTCGACCGCC**---ATGCC**ATG**CTTGCCGAAGTGCTTCGCGACAACGGT |
| P_XR_∆repeat | AG**TTTTTGGGGCCGCTTTGCGGCCCTGGCA**ATGCTGGCGGTCA**ACCTGGCAAAGCCCTGGCTTCGACCGCCCGACCCGGCAAACTGGCAATGACCCGGCA**TATACCG**CATA*CTTG***---***AAAG*GAGC**ATGCC**ATG**CTTGCCGAAGTGCTTCGCGACAACGGT |
| P_XR_-5’perfect | AG**TTTTTGGGGCCGCTTTGCGGCCCTGGCA**ATGCTGGCGGTCA**ACCTGGCAAAGCCCTGGCTTCGACCGCCCGACCCGGCAAACTGGCAATGACCCGGCA**TATACCG**CATA*CTT*TTCGGCAAATGCCGA*AAAG*GAGC**ATGCC**ATG**CTTGCCGAAGTGCTTCGCGACAACGGT |
| P_XR_-3’perfect | AG**TTTTTGGGGCCGCTTTGCGGCCCTGGCA**ATGCTGGCGGTCA**ACCTGGCAAAGCCCTGGCTTCGACCGCCCGACCCGGCAAACTGGCAATGACCCGGCA**TATACCG**CATA*CTTG*TCGGCAAATGCCGAC*AAG*GAGC**ATGCC**ATG**CTTGCCGAAGTGCTTCGCGACAACGGT |

The four repeats in the promoter are indicated by colour (S1, yellow; S2, green; S3, purple; S4, brown) with the repeat underlined and the imperfect palindromic part in italics. Dashes (-) indicate deletions and the start codon of the *xre* gene is shown with a boldface ATG. Substitutions in relation to the wild type sequence (P_XR_, top) are shown with red letters.
