# Supplementary material for: Structural basis for higher-order DNA binding by a bacterial transcriptional regulator
Source: PLoS Genet. 2025 Jun 27;21(6):e1011749. doi: 10.1371/journal.pgen.1011749 (PMC12204516; doi:10.1371/journal.pgen.1011749)
Supplement: S2 Table — (DOCX) [file pgen.1011749.s009.docx]

**S2 Table. Strains and plasmids used in this study**

| **Strains** | **Genotype or description** | **Source** |
| --- | --- | --- |
| MG1655 | *E. coli* K-12 *F– λ– ilvG– rfb-50 rph-1* | Laboratory collection |
| BL21(DE3) | *E. coli* B *F^–^ dcm ompT hsdS*(*rB^–^ mB^–^*) *gal* | Agilent |
| **Plasmids** |  |  |
| pUC57::*xre-res*^Pp^ | *P. putida* KT2440 *xre-res* locus including 227bp upstream/60bp downstream | [1] |
| pBAD33 | p15, *araC*, P_BAD_, Cm^r^ | [2] |
| pBAD33::*xre*_opSD_^Pp^ | *P. putida xre* with an optimized Shine-Dalgarno (opSD)^1^ | This work |
| pBAD33::*xre*_naSD-_*res*^Pp^ | *P. putida xre-res* locus with a native SD (naSD) | This work |
| pBAD33::*xre*_naSD_^R67A^_-_*res*^Pp^ | *P. putida xre*^R67A^*-res* locus with a native SD (naSD) | This work |
| pGH254K | Mini-R1, *lacZYA* transcriptional fusion vector, Kan^r^ | [3] |
| pGH254Kgfp | Mini-R1, *gfp* transcriptional fusion vector, Kan^r^ | This work |
| pGH254Kgfp::P_XR_ | Transcriptional fusion of P_XR_-*xre’* to *gfp* | This work |
| pGH254Kgfp::P_XR_∆1 | Deletion of Sequence 1 in P_XR_ (bp 1-34) | This work |
| pGH254Kgfp::P_XR_∆1-2 | Deletion of Sequence 1-2 in P_XR_ (bp 1-71) | This work |
| pGH254Kgfp::P_XR_∆1-3 | Deletion of Sequence 1-3 in P_XR_ (bp 1-101) | This work |
| pGH254Kgfp::P_XR_∆4 | Deletion of Sequence 4 in P_XR_ (bp 107-137) | This work |
| pGH254Kgfp::P_XR_∆3-4 | Deletion of Sequence 3-4 in P_XR_ (bp 71-137) | This work |
| pGH254Kgfp::P_XR_∆repeat | Deletion of perfect part of repeat in Sequence 4 in P_XR_ (bp 115-129) | This work |
| pGH254Kgfp::P_XR_5’perf | Perfect 5’-repeat in Sequence 4 (substitution G115T) | This work |
| pGH254Kgfp::P_XR_3’perf | Perfect 3’-repeat in Sequence 4 (substitution A130C) | This work |
| pET-29b(+) | *ori*ColE1, Km^r^, T7 promoter | Twist Bioscience |
| pET-29b(+)::*xre*_CHis6_^Pp^ | *P. putida xre* with a C-terminal His_6_-tag | Twist Bioscience |
| pETDuet-1 | Amp^r^, *lacI*, T7 promoter | Novagene |
| pETDuet::*res*_NHis6_-*xre*^Pp^ | *P. putida res* in MCSI, adding N-terminal His_6_-tag, and *xre* in MCSII | This work |
| pETDuet::*xre*_NHis6_-*res*^Pp^ | *P. putida xre* in MCSI, adding N-terminal His_6_-tag, and *res* in MCSII | This work |

The names of the strains and plasmids are listed together with a genotype or description and the source. Detailed information about the plasmid constructions can be found in **Appendix Supplementary Methods**. ^1^The optimized (op) Shine-Dalgarno (SD) sequence (TAAGGAGGAAATTAA) was included in 5’ end of the construct and composed of a strong SD (TAAGGAGG) [4] and additional A’s and T’s (AAATTAA) to optimize translation.

**REFERENCES**

1. Skjerning RB, Senissar M, Winther KS, Gerdes K, Brodersen DE. The RES domain toxins of RES-Xre toxin-antitoxin modules induce cell stasis by degrading NAD. Mol Microbiol. 2018;0: 1–16. doi:10.1111/mmi.14150

2. Guzman LM, Belin D, Carson MJ, Beckwith J. Tight regulation, modulation, and high-level expression by vectors containing the arabinose PBAD promoter. J Bacteriol. 1995;177: 4121–30.

3. Bærentsen RL, Nielsen SV, Skjerning RB, Lyngsø J, Bisiak F, Pedersen JS, et al. Structural basis for kinase inhibition in the tripartite E. coli HipBST toxin-antitoxin system. Elife. 2023;12: 2022.01.28.478185. doi:10.7554/eLife.90400

4. Ringquist S, Shinedling S, Barrick D, Green L, Binkley J, Stormo GD, et al. Translation initiation in Escherichia coli: sequences within the ribosome-binding site. Mol Microbiol. 1992;6: 1219–29.
