# Supplementary material for: Structural basis for higher-order DNA binding by a bacterial transcriptional regulator
Source: PLoS Genet. 2025 Jun 27;21(6):e1011749. doi: 10.1371/journal.pgen.1011749 (PMC12204516; doi:10.1371/journal.pgen.1011749)
Supplement: S3 Table — (DOCX) [file pgen.1011749.s010.docx]

**S3 Table. DNA oligonucleotides used in this study**.

| **Oligonucleotide** | **Sequence (5’-3’)** |
| --- | --- |
| xre_opSD_Fw | ccccaGGTACCTAAGGAGGAAATTAAATGCTTGCCGAAGTGCTTCG |
| xre_Rv | aaaaaGTCGACTCACAGGCCATAGCCCTCG |
| xre_naSD_Fw | AAAACGGTACCcaaatgccgaaaaggagcatgcc |
| res_Rv | AAAAAGTCGACCTAGAACAGGCGGCTGTCC |
| xre(R67A)_Fw | CATCCCGCTGGCGACCCTCAAATCCC |
| xre(R67A)_Rv | ATCTGGTCGCGCTCC |
| pGH254K_gib_Fw | gatgaactatacaaataaatattataaaaattgcctgatacgctgcg |
| pGH254K_gib_Rv | gttcttctcctttactcataagctgtttcctgtgtgataaagaaagt |
| GFPmut2_gib_Fw | ttatcacacaggaaacagcttatgagtaaaggagaagaacttttcactggag |
| GFPmut2_gib_Rv | tatcaggcaatttttataatatttatttgtatagttcatccatgccatgtg |
| pGH254Kgfp_T6613_Fw | AGGAAACAGCtaTGAGTAAAGGAG |
| pGH254Kgfp_T6613_Rv | GTGTGATAAAGAAAGTTAAAATG |
| PXR_Fw | aacccGAATTCagtttttggggccgctttgc |
| PXR_Rv | aaaaaGGATCCaccgttgtcgcgaagcacttc |
| PXR∆S1_Fw | TGGCGGTCAACCTGGCAA |
| PXR∆S1_Rv | GAATTCAGTTTGTAGAAACGCAAAAAGGC |
| PXR∆S1-2_Fw | CGACCCGGCAAACTGGCA |
| PXR∆S1-2_Rv | GAATTCAGTTTGTAGAAACGCAAAAAGGC |
| PXR∆S1-3_Fw | ATACCGCATACTTGTCGG |
| PXR∆S1-3_Rv | GAATTCAGTTTGTAGAAACGC |
| PXR∆S4_Fw | ATGCCATGCTTGCCGAAG |
| PXR∆S4_Rv | CGGTATATGCCGGGTCATT |
| PXR∆S3-4_Fw | ATGCCATGCTTGCCGAAGTGC |
| PXR∆S3-4_Rv | GGCGGTCGAAGCCAGGGC |
| PXR∆repeat_Fw | AAAGGAGCATGCCATGCT |
| PXR∆repeat_Rv | CAAGTATGCGGTATATGCC |
| PXR-5’perf_Fw | CCGCATACTTtTCGGCAAATG |
| PXR-5’perf_Rv | TATATGCCGGGTCATTGC |
| PXR-3’perf_Fw | CAAATGCCGAcAAGGAGCATG |
| PXR-3’perf_Rv | CCGACAAGTATGCGGTAT |
| res_mcsI_Fw | AAACCGGATCCGTGATTTTGTGGCGAATCAGCG |
| res_mcsI_Rv | AAAAAGTCGACCTAGAACAGGCGGCTGTCCG |
| xre_mcsII_Fw | AACCCCATATGatgcttgccgaagtgcttcg |
| xre_mcsII_Rv | AAAAACTCGAGtcacaggccatagccctcg |
| xre_mcsI_Fw | aaaaaGGATCCgcttgccgaagtgcttcgcgac |
| xre_mcsI_Rv | aaaaaGTCGACtcacaggccatagccctcg |
| res_mcsII_Fw | aggggCATATGattttgtggcgaatcagcg |
| res_mcsII_Rv | aaaaaCTCGAGctagaacaggcggctgtcc |
| S1_fwd | TTTTTGGGGCCGCTTTGCGGCCCTGGCA |
| S1_rev | TGCCAGGGCCGCAAAGCGGCCCCAAAAA |
| S2_fwd | ACCTGGCAAAGCCCTGGCTTCGACCGCC |
| S2_rev | GGCGGTCGAAGCCAGGGCTTTGCCAGGT |
| S3_fwd | CGACCCGGCAAACTGGCAATGACCCGGCAT |
| S3_rev | ATGCCGGGTCATTGCCAGTTTGCCGGGTCG |
| S4_fwd | CATACTTGTCGGCAAATGCCGAAAAGGAGC |
| S4_rev | GCTCCTTTTCGGGATTTGCCGACAAGTATG |
| S4_5'perf_fwd | CATACTTTTCGGCAAATGCCGAAAAGGAGC |
| S4_5'perf_rev | GCTCCTTTTCGGGATTTGCCGAAAAGTATG |
| S4_3'perf_fwd | CATACTTGTCGGCAAATGCCGACAAGGAGC |
| S4_3'perf_rev | GCTCCTTGTCGGGATTTGCCGACAAGTATG |
|  |  |
|  |  |
|  |  |
|  |  |

The table includes the names of the oligonucleotides and their DNA sequence.
