# Supplementary material for: Structural basis for higher-order DNA binding by a bacterial transcriptional regulator
Source: PLoS Genet. 2025 Jun 27;21(6):e1011749. doi: 10.1371/journal.pgen.1011749 (PMC12204516; doi:10.1371/journal.pgen.1011749)
Supplement: S1 Text — (DOCX) [file pgen.1011749.s011.docx]

**Structural basis for higher-order DNA binding by a bacterial transcriptional regulator**

*Frederik O. G. Henriksen, Lan B. Van, Ditlev E. Brodersen, and Ragnhild B. Skjerning^*^*

**SUPPORTING INFORMATION METHODS**

**Plasmid constructions**

For pBAD33::*xre*_opSD_, *P. putida* KT2440 *xre* (PP_RS12675) was amplified from pUC57::*xre-res*^Pp^ using primer xre_opSD_Fw, adding an optimized SD (opSD) to the 3’-end, and primer xre_Rv. The resulting PCR product was digested with KpnI and SalI and ligated into pBAD33.

For pBAD33::*xre*_naSD_-*res*^Pp^, *P. putida* KT2440 *xre-res* locus (PP_RS12675- PP_RS12680) was amplified from pUC57::*xre-res*^Pp^ using primer xre_naSD_Fw, including the native SD (opSD) at the 3’-end, and primer res_Rv. The resulting PCR product was digested with KpnI and SalI and ligated into pBAD33.

For pBAD33::*xre*_naSD_^R67A^-*res*^Pp^, substitution of arginine-67 (R67) in Xre with alanine (A) was done using Q5 Site-Directed Mutagenesis Kit from NEB with pBAD33::*xre*_naSD_-*res*^Pp^ as the template and primers xre(R67A)_Fw and xre(R67A)_Rv.

*P. putida* KT2440 *xre-res* locus (PP_RS12675- PP_RS12680) was amplified from pUC57::*xre-res*^Pp^ using primer xre_naSD_Fw, including the native SD (opSD) at the 3’-end, and primer res_Rv. The resulting PCR product was digested with KpnI and SalI and ligated into pBAD33.

For pGH254Kgfp, the *lacZYA* operon from pGH254K was replaced with *gfpmut2* using Gibson Assembly (NEB) according to the protocol. The *gfpmut2* gene encodes a GFP mutant containing a triple substitution; S65A, V68L, and S72A, that confers enhanced fluorescence emission and more efficient folding at 37°C(1). The vector backbone was amplified from pGH254K using primer pGH254K_gib_Fw  and pGH254K_gib_Rv, while the *gfpmut2* insert was amplified from synthetic DNA ordered from Twist Bioscience. Sequencing revealed an insertion (T6613) in the ribosomal binding site (RBS), which was removed using the Q5 Site-Directed Mutagenesis Kit from NEB. Primers pGH254Kgfp_T6613_Fw and pGH254Kgfp_T6613_Rv were designed using NEBaseChanger.

For pGH254Kgfp::P_XR_, The *xre-res* promoter (P_XR_) was amplified from pUC57::*xre-res* using primers PXR_Fw and PXR_Rv. The resulting PCR product was digested with EcoRI and BamHI and ligated into pGH254Kgfp.

For pGH254Kgfp::P_XR_∆S1, deletion of S1 from P_XR_, corresponding to basepair 1-34, was done using Q5 Site-Directed Mutagenesis Kit from NEB with pGH254Kgfp::P_XR_ as the template and primers PXR∆S1_Fw and PXR∆S1_Rv.

For pGH254Kgfp::P_XR_∆S1-2, deletion of S1-2 from P_XR_, corresponding to basepair 1-71, was done using Q5 Site-Directed Mutagenesis Kit from NEB with pGH254Kgfp::P_XR_ as the template and primers PXR∆S1-2_Fw and PXR∆S1-2_Rv.

For pGH254Kgfp::P_XR_∆S1-3, deletion of S1-3 from P_XR_, corresponding to basepair 1-101, was done using Q5 Site-Directed Mutagenesis Kit from NEB with pGH254Kgfp::P_XR_ as the template and primers PXR∆S1-3_Fw and PXR∆S1-3_Rv.

For pGH254Kgfp::P_XR_∆S4, deletion of S4 from P_XR_, corresponding to basepair 107-137, was done using Q5 Site-Directed Mutagenesis Kit from NEB with pGH254Kgfp::P_XR_ as the template and primers PXR∆S4_Fw and PXR∆S4_Rv.

For pGH254Kgfp::P_XR_∆S3-4, deletion of S3-4 from P_XR_, corresponding to basepair 71-137, was done using Q5 Site-Directed Mutagenesis Kit from NEB with pGH254Kgfp::P_XR_ as the template and primers PXR∆S3-4_Fw and PXR∆S3-4_Rv.

For pGH254Kgfp::P_XR_∆repeat, deletion of sequence 3-4 from P_XR_, corresponding to basepair 115-129, was done using Q5 Site-Directed Mutagenesis Kit from NEB with pGH254Kgfp::P_XR_ as the template and primers PXR∆repeat_Fw and PXR∆repeat_Rv.

For pGH254Kgfp::P_XR_-5’perfect, substitution G115T in P_XR_, generating a perfect 5’-repeat in Sequence 4, was done using Q5 Site-Directed Mutagenesis Kit from NEB with pGH254Kgfp::P_XR_ as the template and primers PXR-5’perf_Fw and PXR-5’perf_Rv.

For GH254Kgfp::P_XR_-3’perfect, substitution A130C in P_XR_, generating a perfect 3’-repeat in Sequence 4, was done using Q5 Site-Directed Mutagenesis Kit from NEB with pGH254Kgfp::P_XR_ as the template and primers PXR-3’perf_Fw and PXR-3’perf_Rv.

For pGH254Kgfp::P_XR_-5’flip, substitution 113-TTGTCG-118 to CGACAA in P_XR_, destroying 5’-repeat in Sequence 4, was done using Q5 Site-Directed Mutagenesis Kit from NEB with pGH254Kgfp::P_XR_ as the template and primers PXR-5’flip_Fw and PXR-5’flip_Rv.

For pGH254Kgfp::P_XR_-3’flip, substitution 129-CGAAAA-134 to TTTTCG in P_XR_, destroying 3’-repeat in Sequence 4, was done using Q5 Site-Directed Mutagenesis Kit from NEB with pGH254Kgfp::P_XR_ as the template and primers PXR-3’flip_Fw and PXR-3’flip_Rv.

For pET-29b(+)::*xre*_CHis6_^Pp^, *P. putida* KT2440 *xre* (PP_RS12675) was designed to fuse a C-terminal His_6_-tag directly onto the C-terminal of Xre, followed by a stop codon (*xre*_CHis6_). The construct was custom synthesized by Twist Bioscience and cloned into pET-29b(+) using restriction sites NdeI and XhoI.

For pETDuet::*res*_NHis6_-*xre*^Pp^, *P. putida* KT2440 *xre* (PP_RS12675) was amplified from pUC57::*xre-res*^Pp^ using primer xre_mcsII_Fw and xre_mcsII_Rv. The resulting PCR product was digested with NdeI and XhoI and ligated into MCSII of pETDuet-1 to generate pETDuet::*xre(mcsII)*. Subsequently, *P. putida* KT2440 *res* (PP_RS12680) and was amplified from pUC57::*xre-res*^Pp^ using primer res_mcsI_Fw and res_mcsI_Rv. The resulting PCR product was digested with BamHI and SalI and ligated into MCSI of pETDuet::*xre(mcsII)*.

For pETDuet::*xre*_NHis6_-*res*^Pp^, *P. putida* KT2440 *xre* (PP_RS12675) and was amplified from pUC57::*xre-res*^Pp^ using primer xre_mcsI_Fw and xre_mcsI_Rv. The resulting PCR product was digested with BamHI and SalI and ligated into MCSI of pETDuet-1 to generate pETDuet::*xre(mcsI)*. Subsequently, *P. putida* KT2440 *res* (PP_RS12680) was amplified from pUC57::*xre-res*^Pp^ using primer res_mcsI_Fw and res_mcsI_Rv. The resulting PCR product was digested with NdeI and XhoI and ligated into MCSII of pETDuet::*xre(mcsI)*.

**REFERENCES**

1. Cormack BP, Valdivia RH, Falkow S. FACS-optimized mutants of the green fluorescent protein (GFP). Gene. 1996;173(1):33–8.
