# Supplementary figures and images for: Structural basis for higher-order DNA binding by a bacterial transcriptional regulator
Source: PLoS Genet. 2025 Jun 27;21(6):e1011749. doi: 10.1371/journal.pgen.1011749 (PMC12204516; doi:10.1371/journal.pgen.1011749)

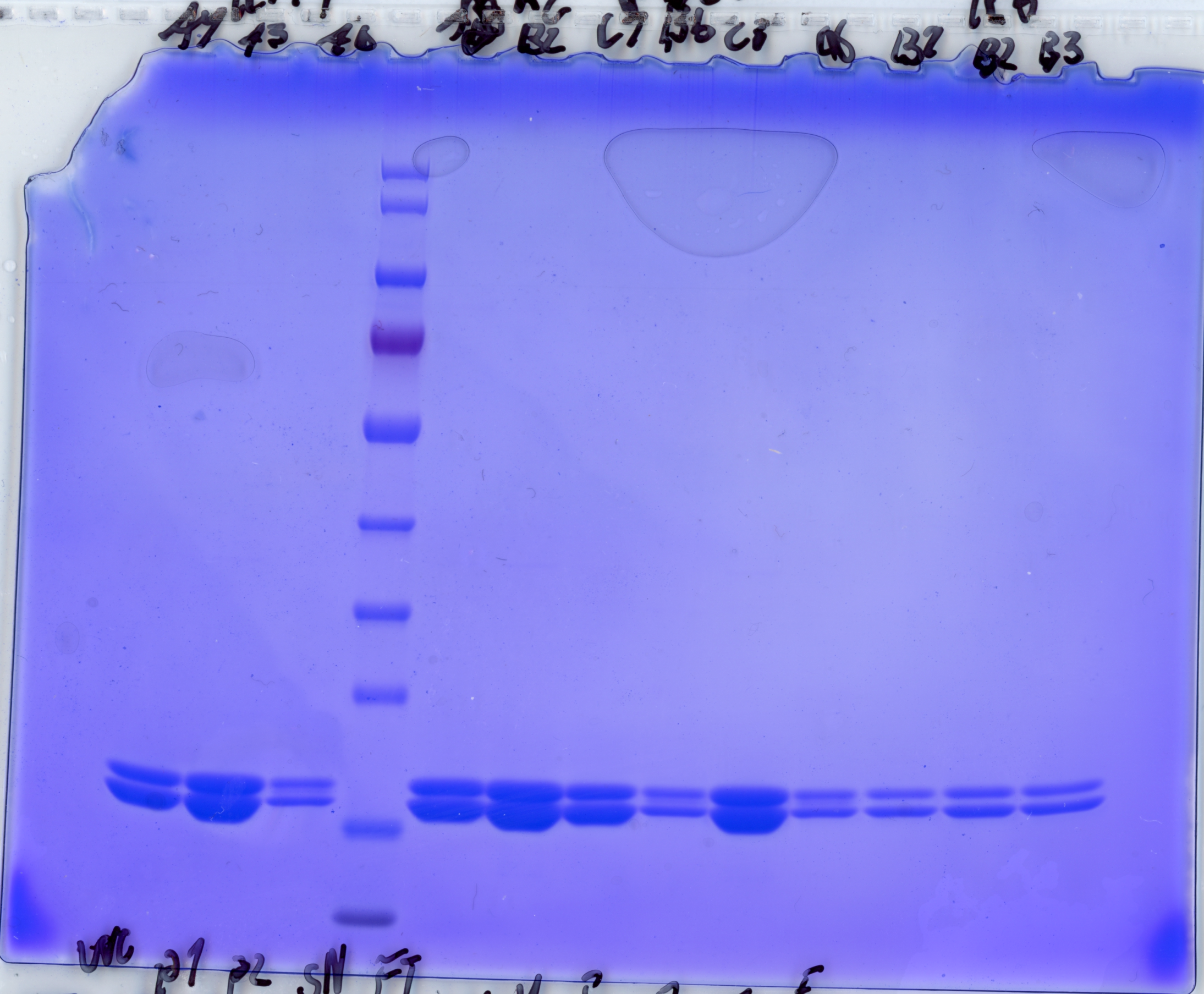

Supplement: S1 Appendix — (ZIP) [file pgen.1011749.s012.zip › Raw data files/Figure S6/Figure S6B.tif]
