# Supplementary material for: Structural basis for higher-order DNA binding by a bacterial transcriptional regulator
Source: PLoS Genet. 2025 Jun 27;21(6):e1011749. doi: 10.1371/journal.pgen.1011749 (PMC12204516; doi:10.1371/journal.pgen.1011749)
Supplement: Attachment — Submitted filename: Response to reviewers.pdf [file pgen.1011749.s015.pdf]

Rebuttal letter: Response to reviewers

Dear Dr. Bager Skjerning.

I am glad to say that the reviewers have commented positively on your revised paper and your manuscript can now be considered as ‘accepted in principle’. However, before I formally accept the paper, please take a close look at the comment provided by reviewer #1. S/he raises an important point regarding Table 1 that I think should be considered.

Best wishes and congratulations on a very nice manuscript,

Kai Papenfort
(Academic Editor)

Reviewers' comments:

Reviewer's Responses to Questions
Reviewer #1: The revisions to the manuscript by Henriksen et al look good except on one point. In my initial review I'd asked the authors to put Ramachandran values into Table 1. The rebuttal says this has been done, but looking at Table 1 in the revision the values are still missing - unless I am missing something? 0.92% Rama outliers (as I can see from edits in MandMs in the marked version) is also a little high. Can you that down any further? Otherwise, looks all fine to me.

We thank the reviewer for noticing this mistake. Table 1 has now been updated with the Ramachandran values. In addition, we have further refined the structure to reduce the number of outliers, which now account for only 0.4%.

Congrats to the authors.

Reviewer #2: Thank you for the authors' reply, which has addressed all of my concerns.

Reviewer #3: Authors have addressed all the comments of the reviewers, but the increment to the quality of the manuscript is minimal, since no additional experimental support was provided.
