# Supplementary material for: Structural basis for higher-order DNA binding by a bacterial transcriptional regulator
Source: PLoS Genet. 2025 Jun 27;21(6):e1011749. doi: 10.1371/journal.pgen.1011749 (PMC12204516; doi:10.1371/journal.pgen.1011749)
Supplement: Attachment — Submitted filename: Response to reviewers.docx [file pgen.1011749.s014.docx]

**Rebuttal letter: Response to reviewers**

Dear Dr. Bager Skjerning.

Thank you for submitting your work to PLOS Genetics. The manuscript has now been reviewed by three experts in the field and their comments are provided below. While the referees find your work potentially interesting, they also raised substantial criticisms. We will consider publishing your manuscript only if you can address these criticisms in a revised version of the manuscript. Of note, one referee indicated that the PDB deposition code and validation summary are missing. Please make sure this is addressed in the revised manuscript.

The structure has now been submitted to the PDB and the accession code + validation report are included with the revised manuscript.

Kind regards,

Kai Papenfort

Reviewer's Responses to Questions

**Comments to the Authors:**
**Please note here if the review is uploaded as an attachment.**

**Reviewer #1:** 
The manuscript from Henriksen et al describes microbiological, biochemical and structural analysis of transcriptional regulation for the Xre-RES toxin antitoxin module from Pseudomonas putida. Whilst having the hallmarks of standard TA systems and transcriptional regulators - TA complexes acting together to negatively autoregulate, use of HTH domains, promoters with palindromic repeats and tandem repeats - the observed phenotypes demonstrate nuances to the regulatory process. These findings demonstrate plasticity in the TA complex itself, use of repeats in the promoter region and also in protein-DNA binding interfaces.

The manuscript was a pleasure to read. Very clear and logical. The experiments are laid out clearly and easy to follow. The conclusions are supported by the presented data and the model looks fair to me. This is a lovely addition to a field that is increasingly showing a depth of diversity and individual specificities to govern biological behaviour.

Thank you for these positive considerations.

I have some suggestions:

Line 119 - the bacteria are transformed, not the constructs.

Corrected.

Line 123 - is there a sentence or two missing? The data in 1D are not discussed (though they clearly show repression)
It is correct that the data in Fig. 1D is not discussed. That is a mistake and has been added to the manuscript.

Line 148 - "of" missing from the end

Corrected.

Line 155 - "the" missing

Corrected.

Line 183 - remove "an"

Corrected.

Fig 4F - The arrangement of the 2x DNAs suggests another go at your SEC and ITC experiments, using an extended DNA probe that goes beyond the 30 bp already used, would likely provide data to support a model of perhaps tighter binding through engagement with a second site. Your discussion implies perhaps you are saving this for the next manuscript so I don't think the experiment should be a requirement here, merely a possible addition.

We acknowledge that this experiment could strengthen our model; however, as the reviewer also implies, we have decided that this is beyond the scope of the present paper.

Table 1 - Please add in ramachandran allowed/outliers etc values

These values were included in the methods section but have now been moved to Table 1.

**Reviewer #2:** 
Frederik Oskar Graversgaard Henriksen and colleagues provide a solid structural and functional characterization of Xre-RES binding to its own promoter to repress transcription. Their study introduces a concentration-dependent model of transcriptional autoregulation by the TA system, supported by X-ray crystallography, size-exclusion chromatography (SEC), AlphaFold3 predictions, and in vivo promoter activity assays.

While the study is well-executed, I have several concerns and suggestions:

1-Promoter activity assays: The authors analyze promoter activity using in vivo GFP assays but do not perform β-galactosidase assays in E. coli, which are a standard approach for measuring transcriptional activity. Was this method considered? Additionally, could the authors clarify why the RFU curves show a decline from initially strong fluorescence intensity? Given the relevance of Pseudomonas putida to the native system, would promoter activity assays in this organism provide additional insights? To complement the fluorescence-based promoter activity assays, a Western blot analysis of GFP protein levels would be beneficial.

We actually believe that our approach adds more information about transcriptional activity than a standard β-galactosidase assay, which is an end-point analysis, as we can follow and quantify expression of GFP in live cells over time. We acknowledge, however, that some readers may be less accustomed to the type of analysis presented.

The initially strong fluorescence observed in all GFP assays is a result of the way the relative fluorescence units (RFU) are calculated. Following overnight growth, bacterial cultures were diluted to a low optical density (OD) of 0.03. At this stage, both the control and measured cultures are expected to have similar OD values and GFP expression levels, theoretically resulting in an RFU close to zero. However, a slight carryover of GFP from the stationary phase (the overnight culture) likely persists in the measured culture. Since fluorescence intensity is normalized to OD, which is very low at this point, even a small difference in fluorescence between the control and experimental cultures leads to a disproportionately high RFU value. Once the cells start to grow, and GFP is not yet expressed, the fluorescence intensity of the measured culture will be reduced and consequently, the RFU values will decrease. This is also one of the reasons why we use a later data point for the bar charts.

Promoter activity assays in *Pseudomonas putida* could in principle provide additional insights by reflecting a more native, biological context. However, we have decided to go with *E. coli* for this experiment, as this model system is close to *Pseudomonas* in many respects and much more well-established in terms of growth and available plasmids.

While we agree that Western blot analysis of GFP protein levels could be useful, these would likely just indicate overall GFP accumulation but not distinguish between newly synthesized and pre-existing protein, as reporter plasmid (pGH254Kgfp) has the promoter region fused to *gfpmut2*, encoding a highly stable GFP variant. We therefore do not think that this experiment would add much, especially given the qualitative nature of Western blots.

2-Direct interaction between Xre-RES and the promoter: While structural data provide strong support for DNA binding, traditional biochemical validation, such as electrophoretic mobility shift assays (EMSA), could further confirm the interaction. Have the authors performed EMSA or similar experiments?

While the structure provides mechanistic insights, we acknowledge that it may not be the most accurate representation of the interaction in solution. Initially, we sought to complement the A-SEC method with EMSA, however, this proved challenging as the addition of a fluorophore to the DNA probe interfered with its interaction with Xre-RES. Consequently, we opted for ITC, which is label-free and offers a more detailed characterization of the interaction.

3-In Figure 4E, the annotation and functional validation: There may be an annotation issue in Figure 4E. Could the authors clarify this? Furthermore, an in vitro mutagenesis assay to assess the essentiality of residue R67 in Xre-RES for the DNA binding activity would strengthen their conclusions.

We thank the reviewer for noticing the mis-annotation in Fig. 4E, which has now been corrected.

We have considered this experiment. The structure suggests that R67 is important for DNA binding, and we support this *in vivo*, by showing that transcriptional repression by *xre^R67A^-res*. However, it is just one of several interacting residues, so the suggested experiment would most likely be inconclusive as a negative result could be interpreted both ways given that R67 might not be essential for the interaction.

4-In Figure 5, when presenting AlphaFold3 predictions, it would be helpful to include confidence metrics such as PTM, iPTM scores. Additionally, a more detailed analysis of predicted interaction interfaces (e.g., hydrogen bonding networks) would improve the interpretation of the structural model.
We agree with the reviewer and have added and the pLDDT plot, as well as PTM and iPTM scores. On the other hand, we do not think that the AF3 model warrants a detailed analysis of the interaction surfaces, as AlphaFold generally has limitation in the ‘correctness’ of its predictions at the rotamer level. We therefore believe that addressing this aspect would require further experimental validation outside of the scope of this publication. The AF model is already strongly validated by the crystal structure, which was determined separately. So, the observation that the key interacting residues are predicted correctly strongly suggests both models are correct.

5-Figure resolution: several figures appear to have low resolution, making it difficult to assess structural details. Higher-quality images would enhance clarity, particularly for structural and interaction data.
We apologize for this problem, which is due to the conversion of the manuscript Word document to PDF. All figures are prepared as high-quality images, and this issue will thus be resolved upon re-submission.

Minor points:

-Figure 1D is not described or interpreted in the manuscript. Could the authors clarify its significance?

We thank the reviewer for this keen observation. It was a mistake that has been corrected in the revised manuscript (line 124-127).

-In figure S5, with the addition of arabinose, the cells appear to grow better. Could the authors explain this phenomenon?

This phenomenon has been observed in all assays of this type. Arabinose is added to induce expression from pBAD33 but is also metabolized by the bacterial cells, providing additional nutrients that enhance growth. The increased growth does not affect the calculated RFU values, since fluorescence intensity of the measured culture is normalized to both the control culture and to OD.

Overall, this study provides valuable mechanistic insights into the transcriptional autoregulation of Xre-RES. Addressing these points would further strengthen the manuscript.

Thank you very much for a very useful review.

**Reviewer #3**: In this manuscript authors study structural basis of DNA binding by a Xre-RES complex. They find that the antitoxin-toxin complex transitions between 2:2 to 4:2 ratios depending on the concentration and that only the 4:2 ratio complex binds the promoter region to repress the transcription of the xre-res genes. In vitro Xre-RES complex binds specifically to S4 element and in vivo likely to a longer region including S3 element. They also conclude that Xre alone is monomeric and highly unstable. RES is therefore required for formation of Xre dimers that can bind DNA. Manuscript is overall well written, however apart from a few novel details, the conclusions are rather expected and similar to those that have been reached for other homologous Xre-RES TAs from other bacteria. Of note, this model of regulation and transition between different order and ratio complexes is conserved for even structurally unrelated TAs, and this is missing in the discussion.
We thank the reviewer for this insightful comment. The term toxin-antitoxin (TA) system covers a large, and highly diverse group of bacterial (mostly) two-gene operons. While we acknowledge that many TA systems are autoregulated by mechanisms that depend on e.g. different ratio complexes, such as conditional cooperativity (which is mentioned in the Introduction), we have decided to focus our discussion on Xre-RES-like homologs. This choice is now clarified in the discussion.

Major remarks:
Line 122 – statement that PXR promoter becomes increasingly active during exponential growth can be questioned – authors used stable GFP protein and the increased signal could be due to progressive accumulation of synthesized GFP protein.

We agree with the reviewer here and this statement was too strongly phrased. From our data, we can only conclude that the promoter is active, which is now reflected in the revised manuscript.

In figure 2 the repeats (operator sites) are discussed and sampled extensively, but there’s no information about the actual promoter – where is it located? Does it overlap with one or few proposed regulation elements? Has it been verified experimentally, for example by 5’ RACE?

Good question, which we have also asked ourselves! Our approach and data allow us to identify regions within the promoter that are important for transcription and its regulation. The precise location of the promoter has not been mapped and we consider it too speculative to draw conclusions based on a predicted location of this.

In figure 2 panel B and D, the first graph with native PXR promoter seems to be the same data based on data points and heights of the bar graphs – if the same data has been plotted (all the experiments performed at the same time?), it should probably be mentioned. Why do the p values differ for these two seemingly identical pairs of bar graphs?

We thank the reviewer for spotting this inconsistency, which occurred as the pairwise tests were accessed through the two-way ANOVA in GraphPad Prism. As the ANOVA assumes equal variance, the estimate is based on all samples included in each analysis, which is erroneous when only doing pairwise comparisons. We have updated our statistical analysis to only use pairwise t-tests between the two compared populations. Based on this, Fig. 2C, 2D, and 5A have been updated.

Figure 4 – it seems like the DNA used for crystallization was not sufficiently long and therefore Xre-RES complex interacts with a DNA from a symmetry-related complex. As authors conclude – only one Xre dimer of two in the complex covers the S4 site, and secondary site inside full operator shall exist. However, the interpretation of the crystallized complex is then difficult, since the discussed distances would likely change in native context. This is partially addressed in comparison to the AF-predicted structure, but authors interpret it as AF-mistake.

We apologize if the AF model was presented as a mistake, this is definitely not how we see it and we have rewritten this part of the discussion to clarify our analysis. In our view, the two structures (AF and Xray) perfectly complement each other and make up for each other's shortcomings. Briefly, while solution-based methods support that the Xre-RES complex interacts with only one copy of the shorter S4 dsDNA, the packing inside the crystal allows the second Xre dimer of the complex to interact with one end of a DNA duplex from a symmetry-related complex, thus hinting at the secondary binding site. We use this observation to support the AlphaFold3 prediction of the interaction with a longer stretch of DNA that provides insights into the interaction of the TA complex with higher-order DNA that would not be evident from experimental data alone. Without the AlphaFold prediction, the secondary DNA interaction would likely have been considered a crystal artifact. And crystallization of the protein complex bound to a longer DNA probe likely very difficult due to the bending of the double helix as most protein-DNA complex crystals depend on end-on stacking of DNA molecules.

Discussion also lacks insights on the dimerization of Xre and the reason for its instability as the monomer. It is likely, that each monomer has different interactions with the toxin, this would bring them close together composing a DNA-binding dimer – this is a bit unusual as compared to stronger antitoxin dimers, stabilized by strand exchange for example in cases of RHH- DNA binding domain.

This complex is very different from "classical TA systems" in which each antitoxin interacts with a cognate toxin in a similar way. Here, because of the 4:2 ratio of antitoxin:toxin, two antitoxins interact directly with a RES toxin while the two other molecules don't interact at all.

However, in contrast to other TA-antitoxins Xre does not seem to have intrinsically disordered regions, and yet curiously it is unstable without its toxin. Are there biochemical or physiological reasons for its instability?

It is a great question that we can only speculate about. Similarly to other TA complexes, an extended part of the antitoxin is extracted and located inside the toxin active site, in this case preventing NAD^+^ binding. However, for Xre it is only 3 residues so not really an unstructured region, however, the conformation of this C-terminus is very different from that of the unbound Xre antitoxin. Whether it be enough to make the isolated antitoxin unstable we cannot say, it could also be due to missing interactions either between the antitoxin molecules or between antitoxin and toxin. We have added our thoughts on this to the discussion. However, without meaningful structural data from an isolated Xre, which we have been unable to achieve, speculation is what we can offer. Based on the model, we believe that the primary interaction between the first Xre and RES will stabilize the structure of Xre. This in turn serves as a nucleation site allowing the second Xre to interact with the Xre_2_-RES_2_ complex forming the Xre_4_RES_2_ complex.

Minor:
Line 203 – incomplete phrase- did authors mean “at different protein:DNA ratios”?

Corrected.

**Have all data underlying the figures and results presented in the manuscript been provided?**
Large-scale datasets should be made available via a public repository as described in the *PLOS Genetics* [data availability policy](https://eur01.safelinks.protection.outlook.com/?url=http%3A%2F%2Ftrack.editorialmanager.com%2FCL0%2Fhttp%3A%252F%252Fjournals.plos.org%252Fplosgenetics%252Fs%252Fdata-availability%2F1%2F010f0195d2f55ffe-41017724-0418-4960-b9ce-94eb9fe5a1a7-000000%2F_CE2vygOmVGrowIErbfw5q9COK-of2id286sYqfXyZM%3D204&data=05%7C02%7Craba%40mbg.au.dk%7C2c45d4860aa74d3932bb08dd6c768988%7C61fd1d36fecb47cab7d7d0df0370a198%7C1%7C0%7C638785978576701893%7CUnknown%7CTWFpbGZsb3d8eyJFbXB0eU1hcGkiOnRydWUsIlYiOiIwLjAuMDAwMCIsIlAiOiJXaW4zMiIsIkFOIjoiTWFpbCIsIldUIjoyfQ%3D%3D%7C0%7C%7C%7C&sdata=lCZFGvC9uQ4B43AVmFioQ4BU942OQuTLvfken2P4vys%3D&reserved=0), and numerical data that underlies graphs or summary statistics should be provided in spreadsheet form as supporting information.

Reviewer #1: Yes

Reviewer #2: None

Reviewer #3: **No:** PDB deposition code and validation summary is missing

PLOS authors have the option to publish the peer review history of their article ([what does this mean?](https://eur01.safelinks.protection.outlook.com/?url=https%3A%2F%2Ftrack.editorialmanager.com%2FCL0%2Fhttps%3A%252F%252Fjournals.plos.org%252Fplosgenetics%252Fs%252Feditorial-and-peer-review-process%2523loc-peer-review-history%2F1%2F010f0195d2f55ffe-41017724-0418-4960-b9ce-94eb9fe5a1a7-000000%2F8w5u5uNkJEJpYST8fj2bekPx1icCXV6B4bmqMTcMWc8%3D204&data=05%7C02%7Craba%40mbg.au.dk%7C2c45d4860aa74d3932bb08dd6c768988%7C61fd1d36fecb47cab7d7d0df0370a198%7C1%7C0%7C638785978576756534%7CUnknown%7CTWFpbGZsb3d8eyJFbXB0eU1hcGkiOnRydWUsIlYiOiIwLjAuMDAwMCIsIlAiOiJXaW4zMiIsIkFOIjoiTWFpbCIsIldUIjoyfQ%3D%3D%7C0%7C%7C%7C&sdata=KvJAfYG3u%2FCUFNyXeQELQo%2BJFXWkJV5LCuyMONhif88%3D&reserved=0)). If published, this will include your full peer review and any attached files.


If you choose “no”, your identity will remain anonymous but your review may still be made public.


**Do you want your identity to be public for this peer review?** For information about this choice, including consent withdrawal, please see our [Privacy Policy](https://eur01.safelinks.protection.outlook.com/?url=https%3A%2F%2Ftrack.editorialmanager.com%2FCL0%2Fhttps%3A%252F%252Fwww.plos.org%252Fprivacy-policy%2F1%2F010f0195d2f55ffe-41017724-0418-4960-b9ce-94eb9fe5a1a7-000000%2Ff4Ud2nPAgn0TYTK3zCRwgt_l6qkcVnu7DBGZubVxeYA%3D204&data=05%7C02%7Craba%40mbg.au.dk%7C2c45d4860aa74d3932bb08dd6c768988%7C61fd1d36fecb47cab7d7d0df0370a198%7C1%7C0%7C638785978576794618%7CUnknown%7CTWFpbGZsb3d8eyJFbXB0eU1hcGkiOnRydWUsIlYiOiIwLjAuMDAwMCIsIlAiOiJXaW4zMiIsIkFOIjoiTWFpbCIsIldUIjoyfQ%3D%3D%7C0%7C%7C%7C&sdata=fUeT9P62TXroRFeRItxS9TF1S1zVgM7FydseUFVVEkk%3D&reserved=0).

Reviewer #1: No

Reviewer #2: No

Reviewer #3: No

[NOTE: If reviewer comments were submitted as an attachment file, they will be attached to this email and accessible via the submission site. Please log into your account, locate the manuscript record, and check for the action link "View Attachments". If this link does not appear, there are no attachment files.]

**Figure resubmission:**

While revising your submission, please upload your figure files to the Preflight Analysis and Conversion Engine (PACE) digital diagnostic tool, <https://pacev2.apexcovantage.com/>. PACE helps ensure that figures meet PLOS requirements. To use PACE, you must first register as a user. Registration is free. Then, login and navigate to the UPLOAD tab, where you will find detailed instructions on how to use the tool. If you encounter any issues or have any questions when using PACE, please email PLOS at [figures@plos.org](mailto:figures@plos.org). Please note that Supporting Information files do not need this step. If there are other versions of figure files still present in your submission file inventory at resubmission, please replace them with the PACE-processed versions.

**Reproducibility:**

To enhance the reproducibility of your results, we recommend that authors of applicable studies deposit laboratory protocols in protocols.io, where a protocol can be assigned its own identifier (DOI) such that it can be cited independently in the future. Additionally, PLOS ONE offers an option to publish peer-reviewed clinical study protocols. Read more information on sharing protocols at <https://plos.org/protocols?utm_medium=editorial-email&utm_source=authorletters&utm_campaign=protocols>
